# Clinical trial registration during COVID-19 and beyond in the African context: what have we learned?

**DOI:** 10.1186/s13063-022-06412-8

**Published:** 2022-06-06

**Authors:** Lindi Mathebula, Sinazo Runeyi, Charles Wiysonge, Duduzile Ndwandwe

**Affiliations:** 1 Cochrane South Africa, South African Medical Research Council, Tygerberg, 7505 South Africa; 2 HIV and other Infectious Diseases Research Unit, South African Medical Research Council, Tygerberg, 7505 South Africa; 3 Department of Global Health, Faculty of Medicine and Health Sciences, Stellenbosch University, Tygerberg, Cape Town, 7505 South Africa; 4 School of Public Health and Family Medicine, Faculty of Health Sciences, University of Cape Town, Observatory, Cape Town, 7925 South Africa

## Abstract

Since the outbreak of COVID-19, many lives have been impacted especially on the African continent which is already fighting the burden of multiple diseases of poverty. However, clinical research has offered hope for treatment and prevention options for this infectious disease. Despite many COVID-19 clinical trials conducted globally, three countries in Africa account for more than 80% of all trials from the continent registered trials in clinical trial registries. This indicates geographic disparity among COVID-19 research in Africa. From the perspective of clinical trial registration, transparency in clinical research and the availability of data became important for making informed decisions to manage the pandemic. Registries serve as a source of planned, ongoing, and completed trials while allowing efficient funding allocation for research that would not duplicate efforts. Additionally, research gaps can be identified, which provide opportunities for collaboration among researchers. Therefore, a critical lesson learnt during this pandemic is that clinical trial registration is important in facilitating the process of tracking changes made to protocols and minimizing publication bias, thereby promoting and advocating for clinical research transparency. Moreover, registration in a clinical trial registry is a condition for publication and allows for trial summary results to be publicly available. Adhering to the principle of results sharing is especially important for the rapidly growing clinical research activities racing to find evidence-based interventions to end the COVID-19 pandemic.

## Introduction

Since the COVID-19 pandemic, there have been numerous clinical research activities to develop new or repurposed vaccines and medicines, and other therapies to fight the scourge of COVID-19 [1]. A growing number of preprint publications and media statements have been used to provide evidence on the efficacy and safety of vaccine interventions without peer-reviewed evidence of clinical trial data [2]. Clinical trials are the cornerstone of biomedical research to generate robust evidence on the safety and effectiveness of treatment and preventative interventions for use in routine clinical care [3]. Clinical trials involve direct participation of individuals who trust the investigators to conduct such trials based on the best available scientific knowledge and ethical practices.

While there is much research taking place globally, clinical trial transparency helps to build trust in research findings which is an important consideration, especially for the novel coronavirus SARS-CoV-2 vaccines and therapeutics [4]. Access to this information enables the public to understand how vaccines and therapeutics have received emergency use authorization thus mitigating some of the challenges such as vaccine hesitancy and building confidence in the research conducted [5]. Participants’ decision to consent and volunteer to be involved in a research study is based on the understanding that their participation will contribute to advances in the body of knowledge which ultimately helps improve treatment outcomes [4, 6]. If clinical research remains unreported, it may lead to doubts in research efforts to curb the spread of diseases such as COVID-19 disease [7]. Furthermore, if the findings are not disseminated to the general public, the agreement between the researcher and the participant can be broken [8].

The International Clinical Trial Registry Platform (ICTRP), launched by the World Health Organization in 2005, is a public platform which brings together trials registered in registries across the world thus providing a single point of access to all the clinical trials registered globally [9]. A clinical trial register is a database in which key administrative and scientific information about planned, ongoing, and completed trials, enough to identify that trial’s existence, is stored [10]. WHO-ICTRP harmonizes and standardizes the efforts of registries worldwide through their Registry Network and provides a searchable portal of all clinical trials to ensure a complete overview of the research being conducted is accessible to all involved in healthcare decision-making in [11]. The African continent has the Pan African Clinical Trials Registry (PACTR) which caters for clinical research conducted in Africa [12].

Clinical trial registries facilitate research integrity by tracking changes made on protocols which further enhances clinical trial transparency [13]. For this reason, clinical trial registration underscores the importance of upholding research integrity to ensure trust between the public and the science community by promoting transparency in the principle of data sharing [4]. One of the latest recommendations by the International Committee of Medical Journal Editors (ICMJE) is an ethical obligation to share clinical trial results responsibly with the public as research participants put themselves at risk when participating in these clinical trials [14]. ICMJE further recommends that results from each clinical trial be made available in the respective clinical trial registry no later than 12 months after the completion date of the trial [15].

## Importance of clinical trial registration and implications of pandemics in the African continent

Clinical trial registries become vital during health emergencies, as they have reliable clinical research records of previous, planned, and ongoing trials to find effective therapies [4, 7]. Furthermore, clinical trial registries can facilitate access to important information such as the summary results of the trial, thus mitigating the challenges of access to trial information during emergencies [9]. It is with this framework that we highlight the importance of clinical trial registry during health emergencies. Recent analysis of data sharing practices in ICTRP among registries has been conducted and found that clinical trial data sharing practices for the COVID-19 clinical trials accessible in ICTRP compared to other disease conditions have not changed [16]. Therefore, this calls for urgent action to data sharing practices, especially in settings with low resources to maximize the use of PACTR as a means to share clinical trial information to aid in making decisions relevant for the African population.

Clinical trial registration is essential in Africa because of resource constraints to conduct research [17]. As a result of a lack of resources, funding to research in Africa comes primarily from outside of the continent [7]. Allocation of financial resources towards research should be done efficiently with an understanding of what is already funded by accessing the publicly available trial records in registries [5]. Funders and Sponsors must utilize the registries to access trial records and understand the results which don’t need to be allocated financial resources to conduct trials that have already been conducted or have shown insignificant results [5]. Similarly, duplicate research should not be encouraged especially in resource-limited settings like the African countries that need the most interventions [9], but rather encourage opportunities for collaboration. COVID-19 pandemic has highlighted the importance of research in Africa to contribute to the availability of health information specific to this continent [18]. African researchers have a vested interest and an understanding of the African context, and thus they need to be at the forefront of clinical research to find solutions for the African context [7].

The data generated from different trial registries can be pooled together to answer meaningful public health questions for example in systematic reviews and descriptive studies rather than being used in isolation, resulting in inconclusive or biased findings [6]. Over and above, clinical trial registries enable researchers to identify knowledge gaps in the literature by learning about the past trials and getting to know their successes and challenges, what worked, and how to avoid research errors [5].

Registration of clinical trials is also essential for ethical review committees and regulatory authorities to determine the appropriateness of studies being reviewed by evaluating the study's harms, benefits, and redundancy [18]. For example, during the Ebola virus outbreak emergency in West Africa, the WHO review ethics committee had accelerated review of protocols that were accessible only because they had been registered with a clinical trial registry [19]. As such, it promotes fulfillment of ethical responsibility to human volunteers-research contributes to medical knowledge. It is, therefore, an ethical and moral responsibility to register clinical trials that are conducted in the African countries [18].

## What have we learned in this pandemic?

The COVID-19 pandemic has created a global health crisis and has deeply impacted almost everyone’s daily lives. Everyone is looking toward clinical research to offer a ray of hope against a worst-case scenario for this outbreak [20]. Notwithstanding the many trials being conducted on COVID-19, some regulatory authorities and decision-makers are faced with overwhelming research presented in media statements and preprints, making it difficult to move with the necessary speed to develop policies [5]. These concerns are further compounded by the misinformation on COVID-19, which leaves the public having to make their own decisions from the untrusted source of information [5].

Most clinical trials as described by Ndwandwe et al. suggest that the registry trial records are not updated [4]. This is particularly true on the availability of results on trials long after these trials are said to be complete by dates listed on the records and this may cause mistrust in clinical trial conducted [4]. This trend is common across registries globally with inconsistent in completion of sections, as stated in a cross-sectional analysis of trial records, particularly the reporting field of the registries [16]. There is heightened urgency concerning who will access the results for COVID-19 trials while the race to find treatments, diagnostics, and vaccines continue [7]. Clinical data transparency becomes the focal point to alleviate concerns and fears about the conduct of these studies.

Therefore, this places clinical trial registries as an enabler of public confidence in clinical trials for new drugs, vaccines, and diagnostics [6]. Furthermore, the availability of results allows decision-makers to decide on treatment options to respond to the pandemic [4, 7]. Some regulations do not mandate clinical trial disclosure for early-phase trials [6]. It would be worthwhile to publish important trial observations in the public domain sooner rather than later, especially in situations like the pandemic [5].

## Limitations to the use of clinical trial registries in Africa

Historically, research has indicated that Africa conducts fewer trials particularly in smaller sample size compared to other regions globally and the trend is similar for COVID-19 trials in Africa compared to other regions [7]. The use of clinical trial registration is seen as an administrative tick box done before the trial commences, rather than maximizing the full benefit of the using the registry as a resource to share data, find opportunities to collaborate, and allow potential research participants to access trial information with the aim of building confidence in the clinical research being conducted. Publicly, clinical trial registries are not widely known or used by the participants or the public. This shows a need for capacity building for African researchers and knowledge translation activities, particularly for African participants and communities who may be or want to be involved in clinical trials taking place in the African countries. Relatively, registry teams should also use the data in the registries to understand limitations in clinical research, thereby creating an avenue for building capacity in clinical research designs. Most of the trials conducted in Africa are not registered in PACTR owing to the sponsor’s registry preference which leads to reduced awareness of PACTR as a sole WHO primary register in Africa which should be legislated in Africa to be the registry of choice to register clinical trials conducted in Africa.

## Conclusion

In conclusion, an opportunity for African researchers to unite and find a solution for Africa exists through the appropriate use of the existing continental clinical trial registry, the Pan African Clinical Trials Registry. Strengthening public health response and clinical governance will require a shift toward data-sharing norms to facilitate the scientific precision needed for the accurate assessment of medical interventions, especially for COVID-19. Research activities should be strengthened through collaborations among African researchers to ensure that research is ethically conducted and not duplicated. The strength of Africa lies in Africans uniting to maximize the limited resources available to their disposal while ensuring that the problems of Africa are solved.

## Data Availability

Not applicable.
